# Sulfated *Laminarin* Polysaccharides Reduce the Adhesion of Nano-COM Crystals to Renal Epithelial Cells by Inhibiting Oxidative and Endoplasmic Reticulum Stress

**DOI:** 10.3390/ph17060805

**Published:** 2024-06-19

**Authors:** Tian-Qu He, Zhi Wang, Chuang-Ye Li, Yao-Wang Zhao, Xin-Yi Tong, Jing-Hong Liu, Jian-Ming Ouyang

**Affiliations:** 1 Department of Urology, The Affiliated Children’s Hospital of Xiangya School of Medicine, Central South University (Hunan Children’s Hospital), Changsha 410007, China; 2 Institute of Biomineralization and Lithiasis Research, College of Chemistry and Materials Science, Jinan University, Guangzhou 510632, China

**Keywords:** sulfated polysaccharide, endoplasmic reticulum stress, apoptosis, crystal adhesion

## Abstract

**Purpose:** Adhesion between calcium oxalate crystals and renal tubular epithelial cells is a vital cause of renal stone formation; however, the drugs that inhibit crystal adhesion and the mechanism of inhibition have yet to be explored. **Methods:** The cell injury model was constructed using nano-COM crystals, and changes in oxidative stress levels, endoplasmic reticulum (ER) stress levels, downstream p38 MAPK protein expression, apoptosis, adhesion protein osteopontin expression, and cell–crystal adhesion were examined in the presence of *Laminarin* polysaccharide (DLP) and sulfated DLP (SDLP) under protected and unprotected conditions. **Results:** Both DLP and SDLP inhibited nano-COM damage to human kidney proximal tubular epithelial cell (HK-2), increased cell viability, decreased ROS levels, reduced the opening of mitochondrial membrane permeability transition pore, markedly reduced ER Ca^2+^ ion concentration and adhesion molecule OPN expression, down-regulated the expression of ER stress signature proteins including CHOP, Caspase 12, and p38 MAPK, and decreased the apoptosis rate of cells. SDLP has a better protective effect on cells than DLP. **Conclusions:** SDLP protects HK-2 cells from nano-COM crystal-induced apoptosis by reducing oxidative and ER stress levels and their downstream factors, thereby reducing crystal–cell adhesion interactions and the risks of kidney stone formation.

## 1. Introduction

Reactive oxygen species (ROS) are highly reactive molecules containing unpaired electrons, generated during normal physiological processes, including superoxide anions, hydrogen peroxide, hydroxyl radicals, and nitric oxide [1]. When the production of ROS surpasses the cellular antioxidant defense mechanisms, it triggers the oxidation of various cellular components, such as DNA, lipids, and proteins. This oxidation can damage the cellular matrix, resulting in oxidative stress, ultimately leading to cellular damage [2,3]. Research indicates a close correlation between the impairment of renal tubular epithelial cells and the early pathological changes associated with kidney stone formation [4]. In particular, COM crystals can induce excessive ROS production within epithelial cells, subsequently causing tubular damage and inflammation [5]. These compromised cells create critical attachment sites, promoting the accumulation and growth of CaOx crystals in the kidney, thereby elevating the risk of stone formation [4].

Mitochondrial damage stands as the primary instigator of cellular oxidative stress [6]. Persistent opening of the mitochondrial permeability transition pore (mPTP) results in an influx of Ca^2+^ and escalated ROS levels. Consequently, mitochondrial swelling and rupture of the outer membrane ensue, culminating in the release of cytochrome *c* and the initiation of cell death [7]. Mitochondrial oxidative stress frequently coincides with endoplasmic reticulum (ER) stress, a cellular response to protein misfolding or unfolding. Prolonged ER stress directly triggers cytoplasmic ROS generation [8], thereby activating apoptosis pathways involving Caspase 12 [9,10] and p38 MAPK [11].

ER stress regulates cell damage induced by CaOx crystals and plays a pivotal role in kidney stone formation [4]. Yang et al. [12] discovered that COM crystals could induce elevated expression of the ER stress marker C/EBP homologous protein (CHOP) and activate Caspase 12 through in vitro and in vivo investigations. Paleerath et al. [13] demonstrated that COM crystals activated p38 MAPK and disrupted the tight junctions of distal renal tubular epithelial cells. Yang et al. [12] further studied the effect of ER stress on crystal–cell interactions, revealing that ER stress resulted in diminished cell viability and influenced the expression of secreted proteins associated with crystal formation, thereby intensifying crystal–cell adhesion. These findings collectively suggest that ER stress augments crystal formation and adhesion.

It has been reported that polysaccharides can inhibit oxidative stress and ER stress and have a protective effect on cells. Mulberry leaf polysaccharides could protect against hydrogen peroxide (H_2_O_2_)-induced oxidative stress injury in the peripheral blood leukocytes of Megalobrama amblycephala by inhibiting ER stress and maintaining mitochondrial function [14]. Polysaccharides isolated from *Amanita caesarea* (ACPS2) appeared to alleviate inflammation in vivo. Proteomics and bioinformatics analyses showed that the therapeutic effect of ACPS2 is achieved through the regulation of oxidative stress-mediated ER stress [15]. Moreover, *Astragalus* polysaccharide demonstrated the capacity to mitigate cardiomyocyte apoptosis by downregulating ER stress pathway-related factors in diabetic cardiomyopathy rats and H9C2 cells stimulated with high glucose [16]. In another study, Huang et al. [17] observed that LBP exerted protective effects against cisplatin-induced apoptosis in ovarian granulosa cells, achieved by attenuating ER stress and regulating protein levels implicated in apoptosis. Zhou et al. [18] conducted research indicating that LBP effectively hindered ER stress-mediated apoptosis by reducing intracellular ROS levels and suppressing the expression of key proteins, such as GRP78, XBP-1, CHOP, Caspase-12, and Caspase-9. These findings suggested a partial reversal of corticosterone-induced pathological changes.

Natural plant polysaccharides exhibit a more favorable biosafety profile and possess anti-colitic effects [19,20]. Chemical modifications can substantially enhance the biological activity of these polysaccharides [21,22]. Biological activities, such as antioxidation [23], anticancer effects [24], and in vitro and in vivo immunomodulation [25], were notably augmented following sulfation.

Laminaria is a traditionally consumed brown seaweed [26]. *Laminarin* polysaccharides constitute the primary active constituents derived from Laminaria [27,28]. Previously, we modified Laminarin polysaccharide to obtain degraded Laminarin polysaccharide (DLP) and sulfated DLP (SDLP), and the –OSO_3_^−^ content of DLP and SDLP were 1.27% and 24.83%, respectively [29]. The present study aims to comparatively investigate the protective effects of *Laminarin* polysaccharide before and after sulfation on HK-2 cells against apoptosis induced by the diminution of nano-COM crystals. This aim is achieved by mitigating oxidative and ER stresses, as well as diminishing interactions between crystals and cells. These findings offer insights into the potential development of novel drugs aimed at controlling stone formation.

## 2. Results

### 2.1. Characterization of Nano-COM and FITC-COM

**Figure 1:**
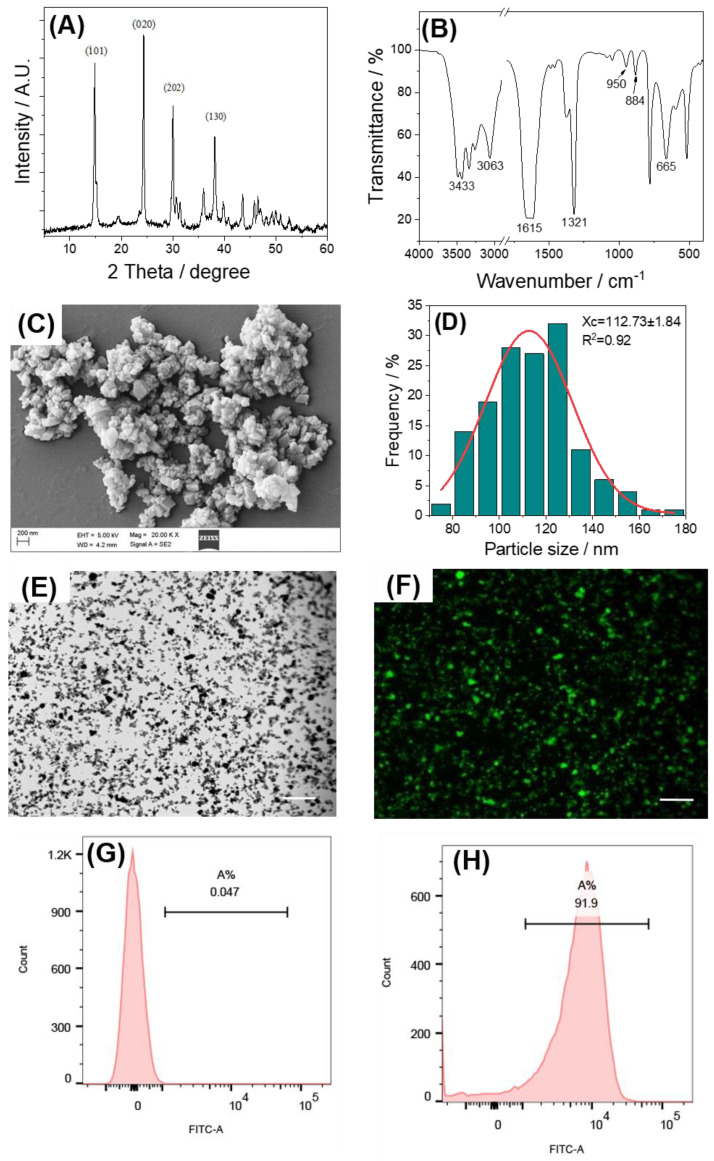
Characterization and fluorescence labeling of Nano-COM crystals. (**A**) XRD spectrum; (**B**) FT-IR spectrum; (**C**) SEM image; (**D**) average COM diameter from the analysis of (**C**) by Nano Measurer 1.2 software; (**E**,**F**) Nano-COM microscopy images before and after FITC labeling; scale bar: 100 μm; (**G**,**H**) fluorescence of Nano-COM before and after FITC labeling detected by flow cytometry.

Figure 1A shows the XRD pattern of the synthesized nano-COM, in which the prominent diffraction peaks located at 2θ = 14.87°, 24.35°, 30.05°, and 38.16° are typical COM diffraction peaks [30] attributed to the ($\bar{1}01$), (020), ($\bar{2}02$), and (130) crystal planes of COM, respectively.

Figure 1B depicts the IR spectrum of the synthesized nano-COM. Absorption peaks within the 3500–3000 cm^−1^ range result from the symmetric and asymmetric stretching vibrations of the O–H single bond in the coordination water molecule [31]. These peaks divide into five absorption peaks with wavelength numbers of approximately 3487, 3433, 3340, 3251, and 3063 cm^−1^, characteristic of COM crystals [32]. The anti-symmetric stretching vibration ν_as_(COO^−^) of the carbonyl group appears at 1615 cm^−1^, while the symmetric stretching vibration ν_s_(COO^−^) is located at 1321 cm^−1^ [33]. Weak absorption of COM crystals in the fingerprint region occurs at around 950 cm^−1^, 884 cm^−1^, and approximately 665 cm^−1^ [34]. The IR spectra further confirm the singular nature of the synthesized COM crystals.

The morphological characteristics of the synthesized nano-COM are shown in Figure 1C. Through Nano measure analysis (Figure 1D), the size was determined to be 86.34 ± 4.39 nm. The crystals exhibit an irregular rhombohedral morphology, with noticeable aggregation attributed to their elevated surface energy.

Fluorescent labeling of nano-COM was executed utilizing FITC. The crystal morphology subsequent to labeling remained consistent with its pre-labeling state (Figure 1E,F). Notably, the labeled nano-COM crystals emitted a vivid green fluorescence. The fluorescently labeled crystals were quantified using flow cytometry, demonstrating a successful labeling rate of 91.9% (Figure 1G,H).

### 2.2. Cytotoxic and Protective Effects of Polysaccharides

**Figure 2:**
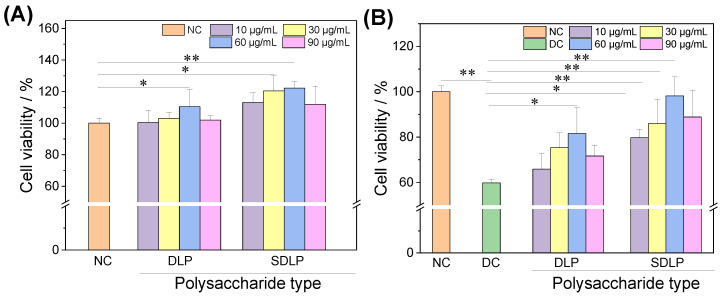
Cytotoxic (**A**) and protective effect on HK-2 cells (**B**) of different concentrations of DLP and SDLP determined by CCK-8 method. NC: normal control group; DC: nano-COM crystal control group, crystal concentration: 200 μg/mL. Protection time: 24 h. Compared with DC group, * *p* < 0.05, ** *p* < 0.01.

Figure 2A shows the cytotoxic effects of varying concentrations of DLP and SDLP on HK-2 cells. The findings reveal that following a 24-h co-culture of cells with DLP and SDLP at concentrations of 10, 30, 60, and 90 μg/mL, the cell viability within each group increased notably, even surpassing the levels of the normal control group by up to 122.26 ± 4.31%. This observation underscores that both DLP and SDLP, within the examined concentration spectrum, do not exhibit toxicity toward HK-2 cells.

Figure 2B shows the protective effects of different DLP and SDLP concentrations (10, 30, 60, and 90 μg/mL) on HK-2 cells. The nano-COM injury group showed 59.74% cell viability, whereas the cell viability of the polysaccharide protection group (65.87–98.19%) was significantly higher than that of the injury group, and the optimal protective concentrations of DLP and SDLP were 60 μg/mL when the cell viability values reached the maximum. At the same concentration, the cell viability of the SDLP-protected group was greater than that of the DLP group.

### 2.3. Reduction in Cellular ROS Levels by Polysaccharides

**Figure 3:**
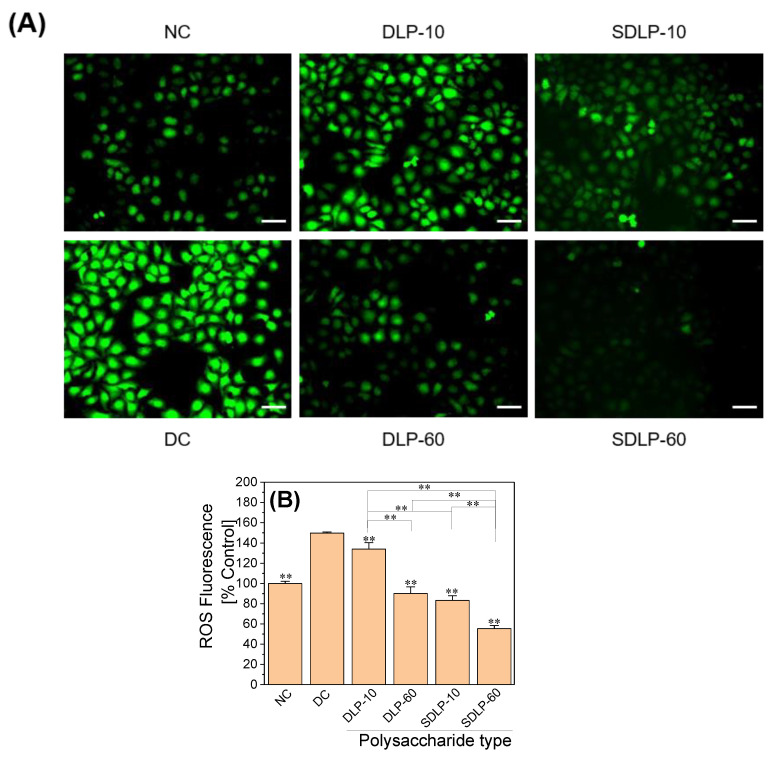
Changes in ROS levels in HK-2 cells before and after protection by different concentrations of DLP and SDLP. (**A**) Fluorescence microscopy; (**B**) columnar quantitative graph. NC: normal control group; DC: nano-COM crystal damage group. DLP-10 and DLP-60 are polysaccharide protection groups, indicating polysaccharide concentrations of 10 μg/mL and 60 μg/mL, respectively. Crystal concentration: 200 μg/mL. Protection time: 24 h. Compared with DC group, ** *p* < 0.01.

Figure 3 shows the changes in cellular ROS in HK-2 cells before and after protection by different DLP and SDLP concentrations. Compared with the control cells, the green fluorescence intensity of the nano-COM damaged group was significantly enhanced (Figure 3A). Under the protection of DLP or SDLP, the green fluorescence intensity of cells was weakened, that is, the ROS level was reduced. Quantitative tests show that the ROS level in cells of the nano-COM-treated group was significantly increased (149.69 ± 1.07%) compared with the normal control group (relative intensity: 100 ± 2.28%) (Figure 3B). In contrast, ROS levels were significantly decreased in the cells of SDLP compared to the DLP-protected group, and the SDLP group exhibited lower ROS levels than the DLP group.

### 2.4. Polysaccharides Reduce Mitochondrial Permeability Transition pore (mPTP) Opening Levels

**Figure 4:**
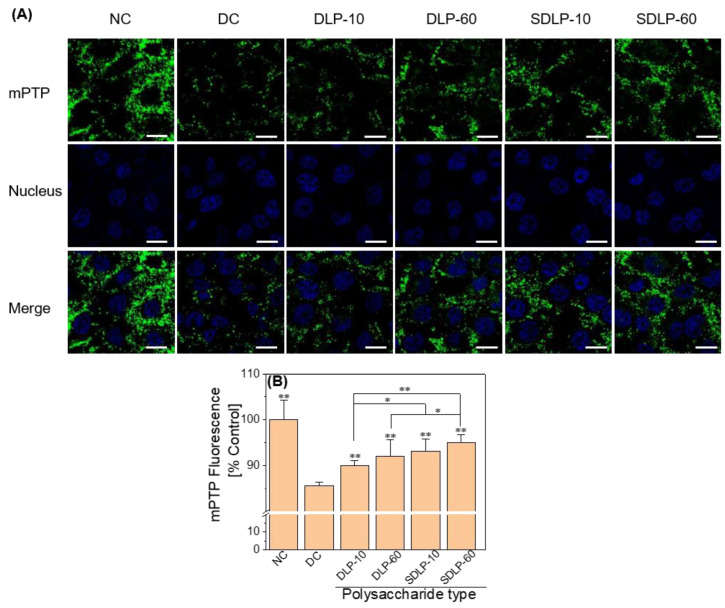
Laser confocal microscopy observation of DLP and SDLP inhibiting nano-COM damage to HK-2 cells to reduce mPTP opening. (**A**) Laser confocal micrograph; (**B**) columnar quantitative graph. NC: normal control group; DC: nano-COM crystal damage group. DLP-10 and DLP-60 are polysaccharide protection groups, indicating polysaccharide concentrations of 10 μg/mL and 60 μg/mL, respectively. Crystal concentration: 200 μg/mL. Protection time: 24 h. Compared with DC group, * *p* < 0.05, ** *p* < 0.01 Scale bar: 20 µm.

The mPTP opening of each group of cells was observed using laser confocal microscopy (Figure 4A). The mitochondria of cells in the normal group demonstrated strong green fluorescence distributed around the nucleus, indicating that the mitochondrial membrane permeability transition pore was at a low opening degree. In contrast, cells in the nano-COM damage group emitted weak and dispersed green fluorescence, indicating that the mPTP was opened. The mPTP opening was inhibited and the mitochondrial green fluorescence in each polysaccharide-protected group was enhanced (Figure 4B), and the order of their fluorescence intensity was: NC > SDLP > DLP > DC, indicating a concentration effect in all of them.

### 2.5. Polysaccharide Reduces ER Stress Level and ER Free Ca^2+^ Ion Concentration

**Figure 5:**
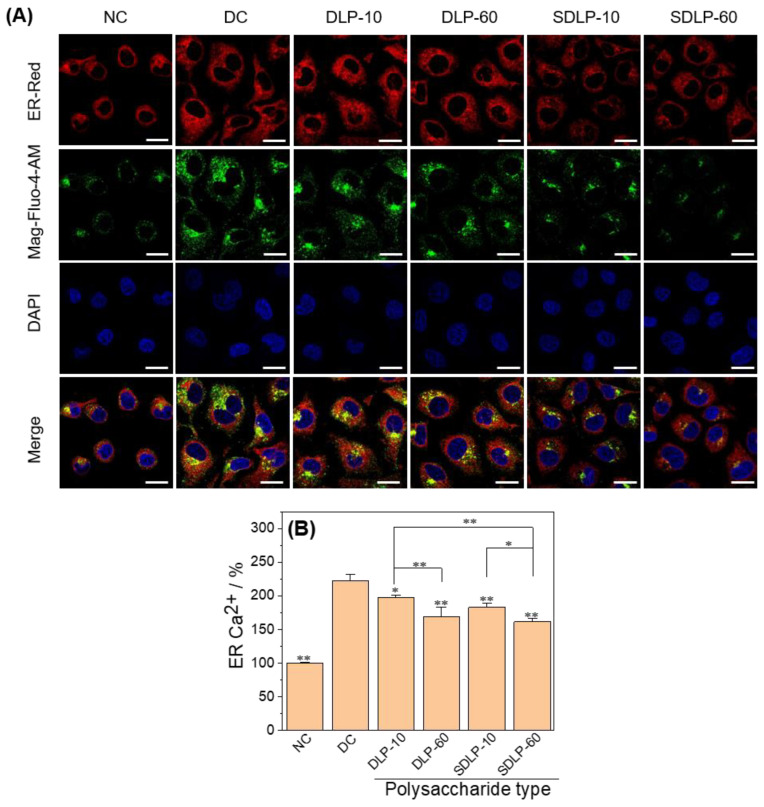
Changes in ERS before and after protection of HK-2 cells by different concentrations of DLP and SDLP. (**A**) Fluorescence micrograph; (**B**) column quantitative graph. The blue fluorescence indicates the nucleus, red fluorescence indicates the endoplasmic reticulum (ER), and green fluorescence indicates the calcium ion concentration in the endoplasmic reticulum (ER Ca^2+^). NC: normal control group; DC: nano-COM crystal damage group. DLP-10 and DLP-60 are polysaccharide protection groups, indicating polysaccharide concentrations of 10 μg/mL and 60 μg/mL, respectively. Crystal concentration: 200 μg/mL. Protection time: 24 h. Compared with DC group, * *p* < 0.05, ** *p* < 0.01. Scale bar: 20 µm.

As seen in Figure 5A, the intensity of ER red fluorescence and green fluorescence reflecting the concentration of Ca^2+^ ions in the ER (ER Ca^2+^) were weak in normal control cells, and the dye was uniformly distributed along the nucleus. In contrast, a brightly dispersed strong red fluorescence of the endoplasmic reticulum and a substantially enhanced green fluorescence of ER Ca^2+^ were observed from cells in the nano-COM damage group, indicating an increase in ER Ca^2+^ concentration. In contrast, the endoplasmic reticulum fluorescence intensity and ER Ca^2+^ fluorescence intensity of the protected group, especially the SDLP group, diminished after pre-protection by polysaccharide.

In Figure 5B, the variations in ER Ca^2+^ concentrations before and after the influence of DLP and SDLP are displayed. Nano-COM-damaged cells exhibited a marked elevation in ER Ca^2+^ levels (221.9%) in comparison to the normal control group (100.0%). Conversely, the polysaccharide-protected group demonstrated varying degrees of reduction in ER Ca^2+^ concentrations (ranging from 161.64% to 197.94%), with the 60 μg/mL SDLP group showcasing the most substantial decline (161.9%)

### 2.6. Polysaccharides Reduce Transcription Factor CHOP Expression

CHOP serves as a transcription factor encoded by the DDIT3 gene, and investigations have substantiated that CHOP stands out as one of the most profoundly upregulated genes during ER stress [18]. CHOP represents a crucial element within the ER stress-induced apoptotic pathway [35]. Huang et al. [36] demonstrated that heightened expression of CHOP can prompt escalated oxidative damage and apoptosis.

**Figure 6:**
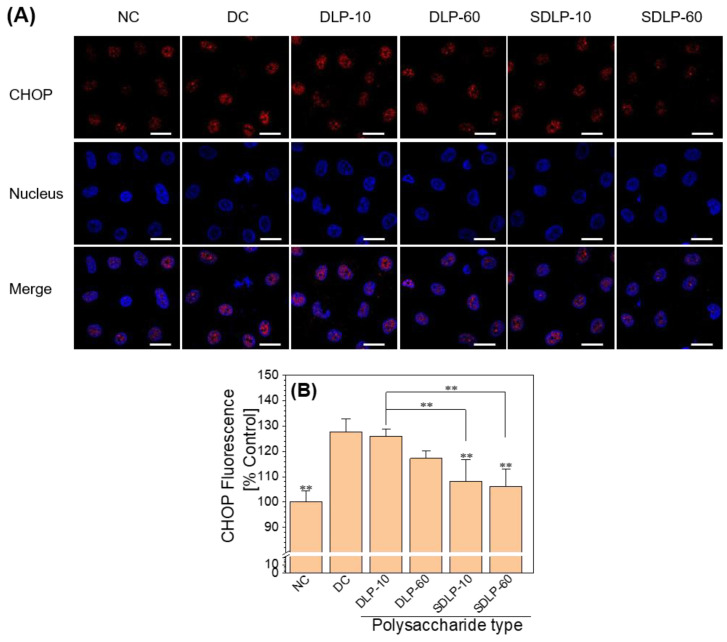
Changes in CHOP expression levels before and after protection of HK-2 cells by different concentrations of DLP and SDLP. (**A**) Fluorescence micrographs; (**B**) quantitative bar graphs. Blue fluorescence indicates nucleus and red fluorescence indicates CHOP. NC: normal control group; DC: nano-COM crystal damage group. DLP-10 and DLP-60 are polysaccharide protection groups, indicating polysaccharide concentrations of 10 μg/mL and 60 μg/mL, respectively. Crystal concentration: 200 μg/mL. Protection time: 24 h. Scale bar: 20 µm. Compared with DC group, ** *p* < 0.01.

As depicted in Figure 6, the expression of CHOP was notably elevated in the group of cells subjected to nano-COM damage when contrasted with the normal control group. Interestingly, in the SDLP group, CHOP expression exhibited a reduction relative to the cells protected by DLP, particularly within the 60 μg/mL DLP-protected subgroup. Notably, the CHOP expression level in this subgroup approached that observed in the normal control group.

### 2.7. Caspase 12 Polysaccharide Reduces Caspase 12 Expression Levels

**Figure 7:**
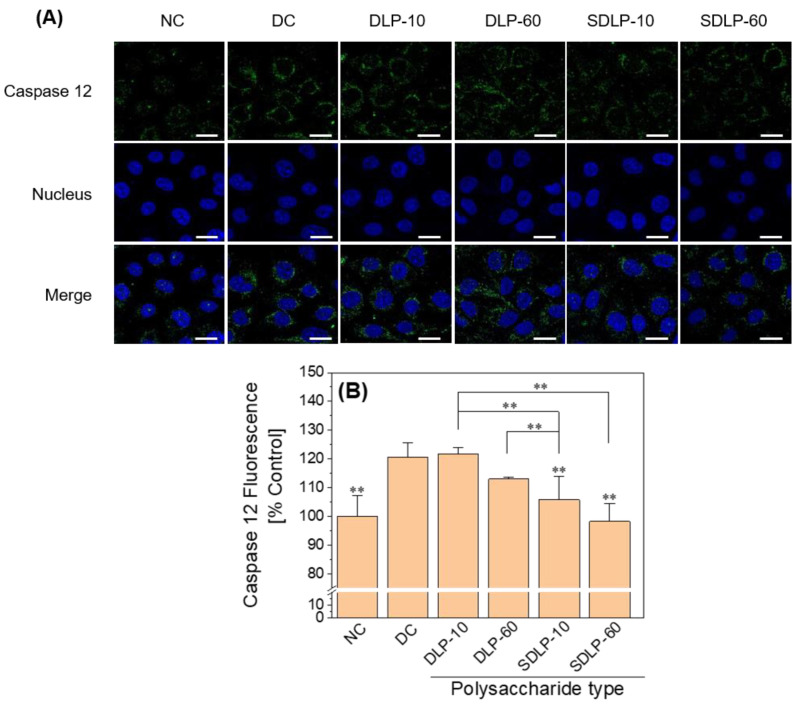
Changes in Caspase 12 expression levels before and after protection of HK-2 cells by different concentrations of DLP and SDLP. (**A**) Qualitative fluorescence plot; (**B**) quantitative bar graph. The blue fluorescence indicates the nucleus and the green fluorescence indicates Caspase 12. NC: normal control group; DC: nano-COM crystal damage group. DLP-10 and DLP-60 are polysaccharide protection groups, indicating polysaccharide concentrations of 10 μg/mL and 60 μg/mL, respectively. Crystal concentration: 200 μg/mL. Protection time: 24 h. Scale bar: 20 µm. Compared with DC group, ** *p* < 0.01.

To delve deeper into the potential mechanism of ER stress-induced apoptosis in HK-2 cells, alterations in Caspase 12, a prominent marker of ER stress, were examined through an immunofluorescence assay [37]. The findings notably unveiled that exposure to nano-COM elicited a substantial rise in Caspase 12 expression. However, the pre-protection by polysaccharides induced a marked reduction in the level of Caspase 12 expression within the cells, as illustrated in Figure 7.

### 2.8. Polysaccharides Reduce the Phosphorylation of p38 (P-p38) Expression Levels

The p38 MAPK serves as a convergence point or shared pathway among various intracellular signaling systems [38]. It is prompted into activation by an array of cellular stresses, encompassing oxidative, genotoxic, and osmotic stressors [39]. Following p38 phosphorylation, the emergence of P-p38 facilitates the direct activation of transcription factors and participation in the body’s stress response [40].

**Figure 8:**
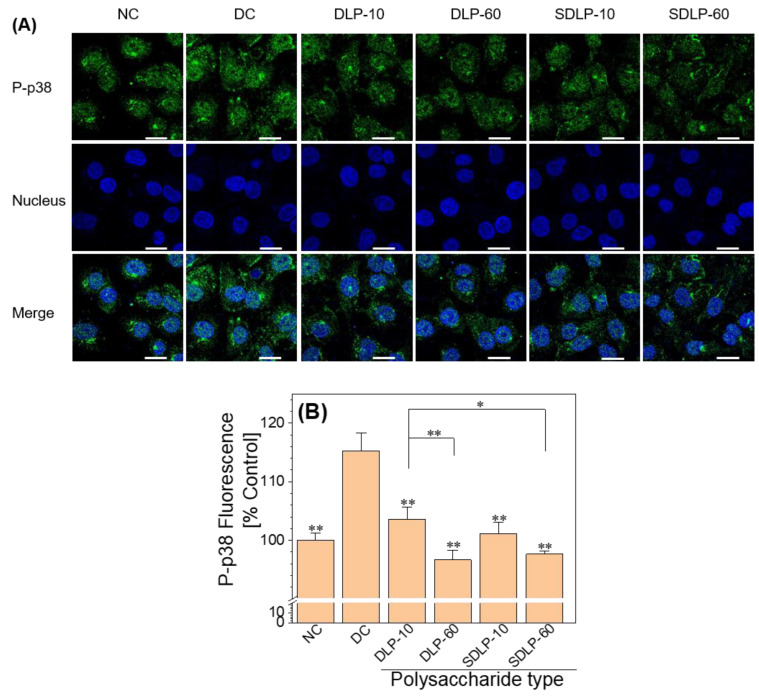
Changes in P-p38 expression levels before and after protection of HK-2 cells by different concentrations of DLP and SDLP. (**A**) Fluorescence micrographs; (**B**) quantitative bar graphs. The blue fluorescence indicates the nucleus and the green fluorescence indicates P-p38. NC: normal control group; DC: nano-COM crystal damage group. DLP-10 and DLP-60 are polysaccharide protection groups, indicating polysaccharide concentrations of 10 μg/mL and 60 μg/mL, respectively. Crystal concentration: 200 μg/mL. Protection time: 24 h. Scale bar: 20 µm. Compared with DC group, * *p* < 0.05. ** *p* < 0.01.

The P-p38 level was assessed via an immunofluorescence assay, as illustrated in Figure 8. In comparison with the normal group (with fluorescence intensity of 100%), the cells subjected to nano-COM injury displayed an increased P-p38 level after 24 h (reaching 115.27%). Conversely, within the polysaccharide-protected group, the level of P-p38 exhibited a reduction (ranging between 96.63% and 103.57%) relative to the aforementioned nano-COM injury scenario.

### 2.9. Qualitative and Quantitative Analyses of Apoptosis Reduction by Polysaccharides

**Figure 9:**
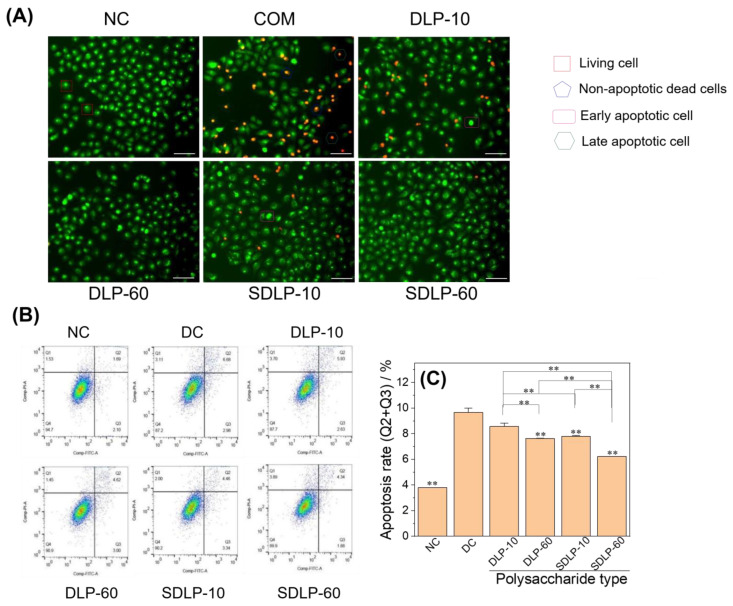
Changes in apoptosis/necrosis before and after protection of HK-2 cells by different concentrations of DLP and SDLP. (**A**) Qualitative assay; (**B**) flow-through quantitative histogram; (**C**) flow-through quantitative histogram. NC: normal control group; DC: nano-COM crystal damage group. DLP-10 and DLP-60 are polysaccharide protection groups, indicating polysaccharide concentrations of 10 μg/mL and 60 μg/mL, respectively. Crystal concentration: 200 μg/mL. Protection time: 24 h. Compared with DC group, ** *p* < 0.01.

Figure 9A displays the fluorescence microscopy images illustrating the changes in apoptosis and necrosis of HK-2 cells before and after protection with varying concentrations of DLP and SDLP. The normal group exhibited a uniform green hue in the cell nuclear chromatin, signifying a normal morphological structure. In stark contrast, the nano-COM damage group revealed spherical or solidified chromatin emitting orange fluorescence, indicative of an advanced apoptotic state. Additionally, some cell nuclei displayed green chromatin with signs of shrinkage or a bead-like appearance, indicating early-stage apoptosis. Notably, within each polysaccharide-protected group, there was a decrease in orange fluorescence and an increase in green fluorescence. Among these, the SDLP group exhibited the most pronounced effect at 60 μg/mL, with cellular status closely resembling that of the normal group cells.

The quantification of apoptotic cells in each group was performed using Annexin V/PI double staining (Figure 9B). The apoptotic rate of cells in the normal group (Q2 + Q3) was 3.79%, notably lower than that observed in the nano-COM damage group (9.66%). Moreover, the apoptosis rate of cells within each polysaccharide-protected group ranged from 6.22% to 8.56%, further underscoring its effectiveness in mitigating apoptosis compared with the crystal damage group (Figure 9C).

The findings depicted in Figure 9 collectively elucidate that the 24-h action of nano-COM crystals on HK-2 cells predominantly induced apoptosis. However, the application of DLP and SDLP exhibited the capacity to inhibit apoptosis. Remarkably, the group protected by SDLP at a concentration of 60 μg/mL demonstrated the most substantial efficacy in this regard.

### 2.10. Polysaccharide Reduces Cell Surface Osteopontin (OPN) Expression

**Figure 10:**
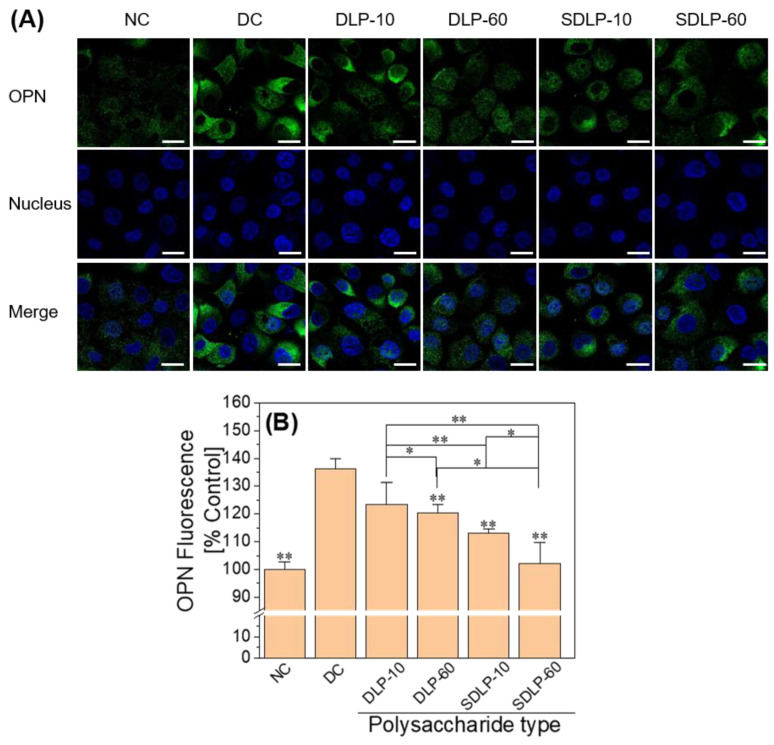
Changes in OPN expression levels before and after protection of HK-2 cells by different concentrations of DLP and SDLP. (**A**) Qualitative fluorescence plot; (**B**) quantitative bar graph. The blue fluorescence indicates the nucleus and the green fluorescence indicates OPN. NC: normal control group; DC: nano-COM crystal damage group. DLP-10 and DLP-60 are polysaccharide protection groups, indicating polysaccharide concentrations of 10 μg/mL and 60 μg/mL, respectively. Crystal concentration: 200 μg/mL. Protection time: 24 h. Compared with DC group, * *p* < 0.05, ** *p* < 0.01. Scale bar: 20 µm.

Figure 10 illustrates the cell surface expression of OPN detected through immunofluorescence staining. In the normal control group, only faint green fluorescence was discernible on the cell surface. In contrast, cells within the nano-COM injury group exhibited notably intensified green fluorescence, indicating a substantial upregulation in the cell surface expression of OPN following injury. Conversely, subsequent protection by DLP and SDLP led to a reduction in green fluorescence on the cell surface, suggesting a diminishment in OPN expression among the protected group.

### 2.11. Qualitative Assay of Polysaccharide to Reduce Crystal Adhesion on Cell Surface

**Figure 11:**
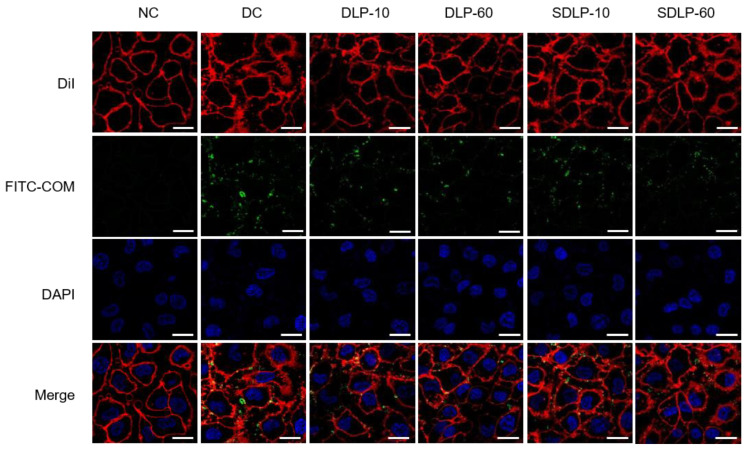
Changes in crystal adhesion on the cell surface before and after polysaccharide protection by laser confocal microscopy. The blue fluorescence is the DAPI-stained nucleus; the green fluorescence is the FITC-labeled nano-COM crystals; the red fluorescence is the DiI-stained cell membrane. NC: normal control group; DC: nano-COM crystal damage group. DLP-10 and DLP-60 are polysaccharide protection groups, indicating polysaccharide concentrations of 10 μg/mL and 60 μg/mL, respectively. Crystal concentration: 200 μg/mL. Protection time: 24 h. Scale bar: 20 µm.

Laser confocal microscopy (Figure 11) was employed to observe the variations in nano-COM crystal adhesion among different groups. In this visualization, FITC-labeled nano-COM crystals emitted a distinct green fluorescence. The findings are evident: the cell surface of the nano-COM damage group exhibited conspicuous green fluorescence, indicating a higher crystal adhesion count on the cell surface. In stark contrast, the cells within the DLP and SDLP protection groups showcased a noteworthy reduction in green fluorescence. This reduction signified a substantial decrease in adhered crystal numbers, with the cell membrane progressively returning to a normal state. Notably, the 60 μg/mL SDLP group exhibited remarkably faint green fluorescence, closely resembling the normal group’s condition.

### 2.12. Quantification of the Proportion of Cells Adhering to the Crystals

**Figure 12:**
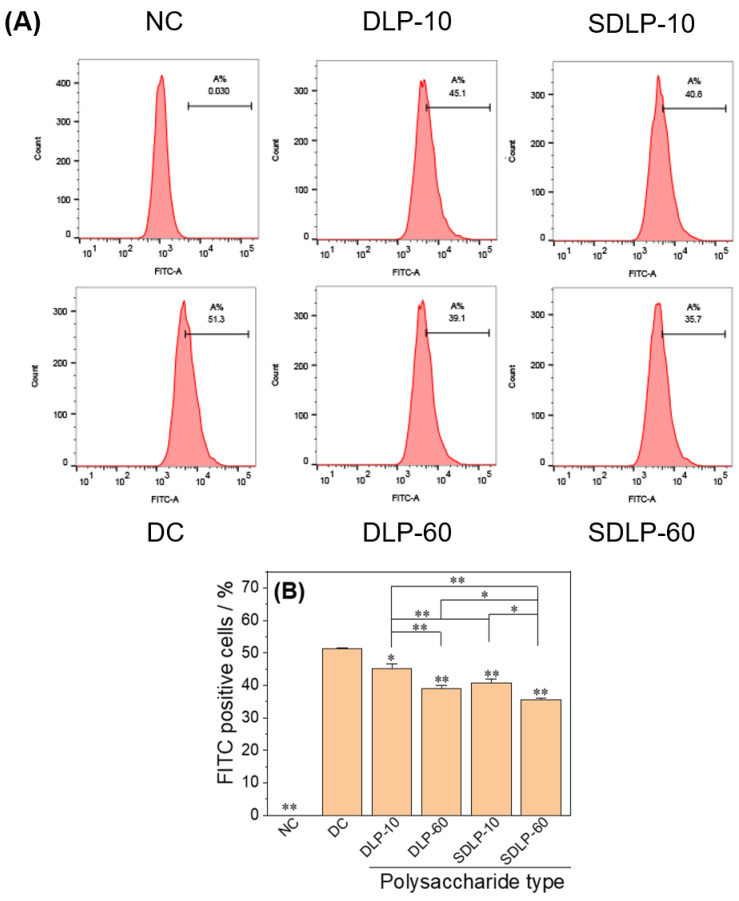
Changes in crystal adhesion on the surface of HK-2 cells before and after protection by different concentrations of DLP and SDLP. (**A**) Flow histogram; (**B**) quantitative bar graph. NC: normal control group; DC: nano-COM crystal damage group. DLP-10 and DLP-60 are polysaccharide protection groups, indicating polysaccharide concentrations of 10 μg/mL and 60 μg/mL, respectively. Crystal concentration: 200 μg/mL. Protection time: 24 h. Compared with DC group, * *p* < 0.05, ** *p* < 0.01.

The extent of nano-COM crystal adhesion to the cell surface was quantitatively assessed using flow cytometry (Figure 12). In the normal control group, crystal adhesion measured a mere 0.03%. However, within the damaged group, this adhesion escalated significantly to 51.3%. Remarkably, within each polysaccharide-protected group, the amount of crystal adhesion decreased to the range of 35.7% to 45.1%. This reduction underscores the enhanced anti-crystal adhesion capacity of the cells following polysaccharide protection.

## 3. Discussion

### 3.1. Characterization of the Laminarin Polysaccharides

In a previous study [29], we degraded the Laminarin polysaccharide to obtain the degraded Laminarin polysaccharide (DLP), and then DLP was sulfated by the sulfur trioxide–pyridine method to obtain the sulfated Laminarin polysaccharide (SDLP). In addition, the FT-IR spectra, GC-MS and 1D and 2D NMR, molecular weight (*M*_w_), and –OSO_3_^−^ content of the polysaccharides were detected.

### 3.2. Nano-COM Causes Mitochondrial Dysfunction and ER Stress by Promoting ROS Overproduction

Under normal physiological circumstances, the organism exerts stringent control over the production of ROS, which acts as a vital mediator governing cell proliferation, protein modulation, transcriptional activity, and their associated functionalities [41]. However, under pathological conditions, when the production of ROS surpasses the cellular antioxidant defense mechanisms [42], it incites oxidative stress. In severe cases, oxidative stress engenders cellular impairment, ultimately culminating in cell demise [43]. Oxidative stress serves as a catalyst for mitochondrial swelling and subsequent disintegration by prompting the opening of the mitochondrial permeability transition pore (mPTP), which consequently induces injury to renal tubular cells. This phenomenon is regarded as the preliminary stage in renal calcium crystallization [44]. Activation of the mPTP disrupts mitochondrial bioenergetic and redox functions, inevitably triggering cell death [45]. Consequently, the pivotal role of mPTP in the cascade of mitochondrial dysfunction leading to cell demise is evident [46]. In this study, we observed that the exposure of HK-2 cells to nano-COM crystals for 24 h led to a marked surge in ROS levels (Figure 3) and a significant upregulation of mPTP opening (Figure 4).

Mitochondria and ER serve as the principal reservoirs for intracellular calcium (Ca^2+^), playing a pivotal role in regulating cellular functions through the transport of Ca^2+^ from the ER to mitochondria [47]. The escalation of ROS production leads to persistent oxidative stress conditions, fostering the accumulation of misfolded and unfolded proteins within the ER lumen, thereby triggering ER stress. Concomitant with ER stress, there is a discharge of Ca^2+^ from the ER into the mitochondria [48], orchestrated by the local cytoplasmic Ca^2+^ concentration (*c* [Ca^2+^]), with a notable effect from Ca^2+^ flux through ER IP3-gated channels and plasma membrane channels facilitating capacitative Ca^2+^ flow [49]. Excessive mitochondrial Ca^2+^ contents incite ROS generation [48]. Pathologically, elevated cellular *c* [Ca^2+^] and oxidative stress-induced conditions can prompt deleterious consequences through mitochondrial Ca^2+^ uptake, potentially culminating in cellular demise [49]. With regard to the ER, the complete and sustained depletion of Ca^2+^ from ER reservoirs, coupled with additional stress cues, may initiate Ca^2+^-dependent forms of apoptosis by eliciting mitochondrial membrane permeability (MMP) [45]. Thus, the excessive ROS generation in renal epithelial cells treated with COM crystals is intrinsically intertwined with ER damage alongside the perturbations to mitochondria. ER stress is characterized by the accumulation of misfolded and unfolded proteins within the ER, the disruption of calcium equilibrium, the activation of unfolded protein responses, ER overload reactions, and Caspase 12-mediated apoptotic pathways [50]. In this study, we demonstrated that Nano-COM crystals substantially reduced ER Ca^2+^ ion levels (Figure 5B) and caused structural disarray within the ER (Figure 5A). Furthermore, Liao et al. [50] observed a pronounced elevation in the expression of ER stress markers, such as GRP78, CHOP, and Caspase 12 in a model of heightened ER stress activation. Our study also unveiled a noteworthy augmentation in the expression of Caspase-12 and CHOP concurrent with the onset of apoptosis (Figure 6 and Figure 7).

### 3.3. DLP and SDLP Inhibit Apoptosis by Regulating ER Stress

Mitogen-activated protein kinases (MAPKs) assume a pivotal role in ER stress-mediated apoptosis [51]. The p38 MAPK signaling pathway can be triggered by COM crystals [52,53]. Koul et al. [52] elucidated that exposure of cells to COM crystals prompted rapid and robust phosphorylation and activation of p38 MAPK, coinciding with DNA synthesis reinstatement. Chaturvedi et al. [53] similarly revealed that oxalate exposure rapidly induced potent p38 MAPK phosphorylation and activation. Liu et al. [54] further illuminated the involvement of p38 phosphorylation (P-p38) in the ER stress-induced apoptosis pathway. Wei et al. [55] expanded these insights, demonstrating that p38 mediates both the ER stress and mitochondrial death pathways, orchestrating early apoptosis in MSCs. Consistent with these findings, our study yielded congruent outcomes—nano-COM crystals were shown to elevate the expression of p38 MAPK downstream of ER stress (Figure 8), culminating in apoptosis onset (Figure 9). Notably, this trajectory was effectively counteracted by DLP and SDLP, with SDLP exhibiting superior inhibition.

Investigations into apoptosis have underscored the multifaceted role of stress-activated p38 MAPK, operating in a context-dependent and cell-type-specific manner. It converges various signals through transcription-dependent and -independent mechanisms, ultimately converging onto caspase activation [56]. Caspases, the initiators of cell death, can be activated through either the extrinsic or intrinsic pathway. The former entails cell surface death receptors activated by corresponding ligands, while the latter is governed by pro-apoptotic Bcl-2 family proteins, modulating mitochondrial permeabilization by releasing proteins from the outer mitochondrial membrane [57]. Notably, cytochrome *c* release from the outer mitochondrial membrane is a pivotal event in the intrinsic apoptotic pathway. Within this cascade, several Bcl-2 family proteins, spanning pro-apoptotic and anti-apoptotic categories, are regulated transcriptionally and post-transcriptionally by the p38 MAPK cascade [56]. For instance, RTMG132-induced apoptosis is channeled through ER stress initiation, prompting the ensuing activation of mitochondria-dependent and -independent Caspase cascade responses [58].

### 3.4. DLP and SDLP Polysaccharides Inhibit Crystal Adhesion on the Cell Surface by Reducing the Expression of Adhesion Molecules

OPN is a glycosylated protein with a negative charge, widely distributed across various tissues and cells. Its role encompasses the regulation of biomineralization in normal and pathological contexts [59]. Among the notable pathological mineralization scenarios is renal stone disease, where OPN’s significance shines prominently [60]. The roster of stone matrix constituents encompasses urinary bridging protein, nephrocalcin, thrombospondin-activated fragment 1, Tamm-Horsfall glycoprotein, Hess B, and OPN [61]. OPN stands out as a major presence within the core, lamellae, and organic matrix of kidney stones in the renal cavity [62]. The secreted OPN and its integration into the kidney stone matrix influence crystal nucleation, growth, aggregation, and adhesion, constituting a crucial element in the morphological transformation of stones, a central process in kidney stone formation [63]. Our investigation also confirmed that OPN expression experienced a significant upregulation at the onset of cell injury (Figure 10).

Diminishing crystal adhesion on cell surfaces is an imperative strategy to thwart stone formation [64]. A prior study from our laboratory [65] revealed that damaged HK-2 cells, upon repair by polysaccharides, showcased marked reductions in OPN expression. The same trend was replicated in this study: polysaccharide protection led to substantially reduced adhesion of nano-COM crystals on cell surfaces, with the 60 μg/mL SDLP-protected group exhibiting the lowest adhesion. This decrease in exposed, negatively charged OPN adhesion molecules curtails the absorption of Ca^2+^ ions from the solution and curbs the adherence and aggregation of positively charged COM crystals on cell surfaces [66]. Furthermore, polysaccharides can adhere to the COM crystal surface, barricading their adhesion to cells [67], thereby mitigating crystal-induced cellular damage. Verkoelen et al. [68] unearthed that natural glycosaminoglycans and semisynthetic polysaccharides (SSPs) emerged as effective inhibitors of crystal adhesion. In our earlier investigation [69], we discerned that polysaccharides could notably amplify the absolute value of zeta potential on the nano-COM crystal surface. This augmentation largely stems from polysaccharides rich in anionic groups adhering to the crystal surface, thus enhancing its negative electrical characteristics [70]. Ultimately, this propels increased repulsion between crystals and cells, contributing to the inhibition of crystal adhesion and aggregation [71]. The effect of polysaccharides on crystal adhesion hinges on their coverage of the crystal surface, the alteration of zeta potential, and their modulation of the crystal–cell receptor interaction [72].

The main limitation of this study is the use of a cell line (HK-2) and no further confirmation of observations in primary cells due to the limited access to the latter. In order to obtain more accurate experimental data and more stable experimental results, it is necessary to use a variety of cell lines for biological activity characterization, combined with primary cells and animal experiments. In addition, we will also combine more quantitative and qualitative analyses with fluorescence-activated cell sorting analysis (FACS) and Western blot techniques to make our study more convincing in the future.

## 4. Materials and Methods

### 4.1. Materials and Equipment

**Materials:***Laminarin* polysaccharide (LP) was produced by Shaanxi Ciyuan Biological Co., Ltd. (Xi’an, China) with *M*_w_ of 23.58 ± 0.44 kDa and –OSO_3_^−^ content of 0.93%. Degraded Laminarin polysaccharide (DLP) and sulfated DLP (SDLP) were obtained according to our previous work [29]. Fluorescein isothiocyanate (FITC) was purchased from Shanghai Macklin Biochemical Co., Ltd. (Shanghai, China). 3-aminopropyltriethoxysilane (APTES) was purchased from Shanghai Aladdin Biochemical Technology Co., Ltd. (Shanghai, China). 4′,6-diamidino-2-phenylindole (DAPI) fluorescent dye, DiI (cell membrane red fluorescent probe), and 4% paraformaldehyde fixative were purchased from Beyotime Biotechnology Co., Ltd. (Shanghai, China).

Human kidney proximal tubular epithelial cells (HK-2) were provided by the Shanghai Cell Bank, Chinese Academy of Sciences (Shanghai, China). Fetal bovine serum, double antibodies, and the DMEM/F12 medium were purchased from Gbico Biochemical Products Ltd. (Grand Island, NY, USA); 6-well plates, 96-well plates, and confocal dishes were purchased from Wuxi NEST Biotechnology Co., Ltd. (Wuxi, China). The Annexin V-FITC/PI double-stained apoptosis assay kit and endoplasmic reticulum red fluorescent probe (ER Red) were purchased from KeyGen Biotech Co., Ltd. (Nanjing, China). Mag-Fluo-4 AM (AAT Bioquest, Pleasanton, CA, USA). Osteopontin (OPN) primary antibody and sheep serum were purchased from Wuhan Boster Biological Technology Co., Ltd. (Wuhan, China). Conjugated Goat anti-rabbit IgG Antibody and iFluor™ 594 Conjugated Goat anti-rabbit IgG Antibody were purchased from Hangzhou HuaAn Biotechnology Co., Ltd. (Hangzhou, China).

**Equipment:** An FTIR spectrometer (Nicolet 6700, Thermo Fisher Scientific, Waltham, MA, USA), X-ray diffractometer (Miniflex 600, Rigaku, Akishima, Japan), field emission scanning electron microscope (Ultra 55, ZEISS, Oberkochen, Germany), full-featured enzyme labeler (Synergy H1, BioTek, Winooski, VT, USA), flow cytometer (FACSCanto, BD, Franklin Lakes, NJ, USA), live cell workstation (Cell Observer, ZEISS, Oberkochen, Germany), and laser confocal scanning microscope (LSM 510 META, ZEISS, Oberkochen, Germany) were used.

### 4.2. Experimental Methods

#### 4.2.1. Synthesis and Characterization of Nano-COM and FITC-Nano-COM Crystals

The nano-COM with a size of approximately 100 nm was synthesized with reference to the literature [73]. First, 50 mL of the 0.60 mol/L CaCl_2_ solution and 50 mL of the 0.60 mol/L Na_2_Ox solution were mixed rapidly at 25 °C and reacted at 1250 rpm/min for 6 min. After centrifugation, the crystals were separated and washed twice with anhydrous ethanol via sonication. After being dried, the target crystals were characterized by FT-IR, XRD, and SEM.

The fluorescent labeling of nano-COM was performed with reference to Zhao et al. [74] with slight modifications. First, 0.05 g of pre-synthesized nano-COM was stirred continuously with 5 mL of 3-aminopropyltriethoxysilane (APTES) in anhydrous ethanol (50 mL) at 74 °C for 3 h. Subsequently, 0.025 g of FITC was added to the above-mentioned mixed system and stirring was continued for 3 h. The COM with FITC labeling was then collected by centrifugation and washed with anhydrous ethanol. The fluorescently labeled crystals were then observed under a fluorescence microscope and the proportion of fluorescently labeled crystals was quantified using flow cytometry.

#### 4.2.2. Cell Culture

A DMEM/F12 culture containing 10% fetal bovine serum and 1% penicillin-streptomycin antibiotics was used to culture HK-2 cells at 37 °C with 5% CO_2_ saturated humidity. When the cells reached 80–90% confluence, the culture medium was discarded, the cells were washed with PBS and 0.25% trypsin-EDTA (Gbico, Grand Island, NY, USA), then the cells were digested in an incubator at 37 °C for 2–3 min, and the digestion degree was observed under a microscope. When cytoplasmic retraction was found and cell space increased, the DMEM/F12 culture medium with 10% fetal bovine serum was added immediately to terminate digestion. The cells were blown to form a cell suspension. After centrifugation, the supernatant was discarded and blown into cell suspension by adding DMEM/F12 containing 10% fetal bovine serum, and an aliquot of the cell suspension was inoculated into the corresponding culture plate to make the cells grow for subsequent experiments.

#### 4.2.3. Cell Viability Detected by CCK-8

##### Cytotoxic Effect of Polysaccharides

Cells were inoculated in 96-well plates at 1.0 × 10^5^ cells/mL, 100 μL/well, and incubated in a 37 °C incubator with 5% CO_2_ for 24 h. Experiments were divided into two groups as follows: (1) normal group: serum-free culture medium was added; (2) polysaccharide group: 10, 30, 60, and 90 μg/mL of *Laminarin* polysaccharide (DLP or SDLP) was added and incubated for 24 h. Three replicate wells were set up for each experiment. After reaching the action time, 10 μL of the CCK-8 reagent was added to each well and incubated for 1 h at 37 °C protected from light, and the OD values were detected at a wavelength of 450 nm using an enzyme marker. The cell viability was obtained using the following equation:

$$Cell\;viability\;(\%)=\frac{OD\;value\;of\;treatment\;group}{OD\;value\;of\;control\;group}\times 100$$

##### Cell Protection by Polysaccharides

Cell density and cell seeding plate were the same as Section Cytotoxic Effect of Polysaccharides. Experiments were divided into three groups: (1) normal group: serum-free culture medium was added; (2) damaged group: 200 μg/mL nano-COM was added and damaged for 24 h; (3) polysaccharide-protected group: 10, 30, 60, and 90 μg/mL of DLP or SDLP mixed with 200 μg/mL nano-COM was added and incubated for 24 h. Three replicate wells were set up for each experiment. After reaching the action time, 10 μL of the CCK-8 reagent was added to each well. After detecting the OD values, the cell viability was obtained using the following equation:

$$Cell\;viability\;(\%)=\frac{OD\;value\;of\;treatment\;group-OD\;value\;of\;Blank\;group}{OD\;value\;of\;control\;group-OD\;value\;of\;Blank\;group}\times 100$$

#### 4.2.4. Polysaccharide Inhibits Oxidative Stress

##### DCFH-DA Staining to Detect Cellular Reactive Oxygen Species (ROS) Levels

Cells were inoculated in 6-well plates at 1.0 × 10^5^ cells/mL, 1 mL/well. After incubation for 24 h, the experiment was divided into the following three groups: (1) normal group: serum-free culture medium was added; (2) damage group: 200 μg/mL of nano-COM crystals were added and damaged for 24 h; (3) polysaccharide protection group: 10 or 60 μg/mL of DLP or SDLP mixed with 200 μg/mL of nano-COM crystals was added to the suspension and incubated for 24 h. After reaching the action time, the supernatant was discarded and 1 mL of the H_2_DCFDA (1:1000) working solution was added to each well and incubated for 30 min at 37 °C protected from light, followed by washing the cells twice with serum-free culture solution and observing them under an inverted fluorescence microscope. Fluorescence semi-quantitative analysis of ROS was performed using ImageJ software (https://doi.org/10.1038/nmeth.2089).

##### Mitochondrial Permeability Transition Pore (mPTP) Assay

The cells were inoculated at the same density as in Section DCFH-DA Staining to Detect Cellular Reactive Oxygen Species (ROS) Levels, inoculated in confocal dishes at 37 °C with 5% CO_2_ for 24 h. After reaching the incubation time, the culture medium was aspirated and the cells were washed with PBS. One milliliter of the fluorescence quenching working solution (prepared from the Calcein AM staining solution and CoCl_2_) was added to each well and incubated for 30 min at 37 °C, protected from light. After incubation, the cells were replaced with a fresh pre-warmed culture medium and incubated for another 30 min. The cells were washed twice with PBS, fixed with 4% paraformaldehyde for 15 min, washed with PBS, and incubated with DAPI staining solution for 5 min at room temperature, followed by observation under laser confocal microscopy.

#### 4.2.5. Polysaccharide Inhibits ER Stress

##### Detection of ER Stress

The seed plates and experimental groups are the same as those in Section Cell Protection by Polysaccharides. After reaching the incubation time, the cells were incubated with 5 μM Mag-fluo-4 AM for 30 min at 37 °C. ER Red dyeing solution (1:1500) pre-warmed at 37 °C was added and incubated at 37 °C for 30 min. After the incubation time was reached, the staining solution was removed, the cells were washed with PBS, and the cells were fixed with 4% paraformaldehyde for 15 min. The cells were washed with PBS three times for 5 min each time, and the DAPI staining solution was added and incubated at room temperature for 5 min. They were then observed under a confocal laser microscope.

##### Fluorescence Detection of Calcium Ion Concentration in the Endoplasmic Reticulum

The experimental grouping is the same as in Section Cell Protection by Polysaccharides. Cells were aspirated after reaching the culture time and PBS was used to wash the cells. Next, 5 μM Mag-Fluo-4 AM were added to the cells and they were incubated at 37 °C for 30 min. Three double holes were set in each group, and the absorbance value was measured at a wavelength of 525 nm.

##### Immunofluorescence Staining and Imaging of CHOP

The seed plates and experimental groups are the same as in Section DCFH-DA Staining to Detect Cellular Reactive Oxygen Species (ROS) Levels. After reaching the incubation time, cells were washed with pre-cooled PBS, fixed with 4% paraformaldehyde for 30 min, washed twice with PBS, and permeabilized with 0.1% Triton X-100 for 15 min. After being washed twice, the cells were closed with 5% BSA for 1 h at room temperature, followed by the addition of CHOP primary antibody (1:800) at 4 °C. The cells were incubated overnight, washed three times with PBS, incubated for 1 h at 37 °C with iFluor™ 594 secondary antibody, washed three times with PBS, and incubated for 5 min at room temperature with DAPI staining solution, followed by the observation of CHOP expression under a laser confocal fluorescence microscope.

##### Immunofluorescence Staining and Imaging of Caspase 12

The seeding plates and experimental grouping are the same as in Section DCFH-DA Staining to Detect Cellular Reactive Oxygen Species (ROS) Levels. After the incubation time was reached, the old medium was removed and the cells were washed with pre-cooling PBS, fixed with 4% paraformaldehyde at room temperature for 15 min, washed with ice PBS 3 times, and permeated with 0.25% Triton X-100 for 15 min. After washing the cells with PBS for 3 × 5 min, the cells were closed with sheep serum for 1 h, and the Caspase 12 primary antibody (1:200) was added and incubated at 4 °C overnight. After the cells were washed with PBS for 3 × 5 min, iFluor™ 594 secondary antibody was added to avoid light, and the cells were incubated at 37 °C for 1 h, washed with PBS for 3 × 5 min, DAPI staining solution was added to avoid light, and they were incubated for 5 min. The expression of Caspase 12 was observed by laser confocal fluorescence microscopy.

##### Immunofluorescence Staining and Imaging of P-p38

The seeding plates and experimental grouping are the same as in Section DCFH-DA Staining to Detect Cellular Reactive Oxygen Species (ROS) Levels. After the incubation time was reached, the culture medium was aspirated and the cells were washed with pre-cooled PBS and fixed at room temperature for 30 min with 4% paraformaldehyde. After washing the cells twice, the cells were permeabilized with 0.25% Triton X-100 for 30 min, washed twice, and sealed with sheep serum for 1 h. P-p38 primary antibody (1:1000) was added and incubated overnight at 4 °C, cells were washed with PBS for 2 × 5 min, iFluor™ 488 secondary antibody was added in dark light, cells were incubated at 37 °C for 1 h, cells were washed with PBS for 2 × 5 min, and then DAPI staining solution was added and incubated in dark light for 5 min. The expression of P-p38 was observed by laser confocal fluorescence microscopy.

#### 4.2.6. Polysaccharide Inhibited the Adhesion of Nano-COM Crystal

##### Detection of Cell Surface Osteopontin (OPN) Expression

The seed plates and experimental groupings were the same as in Section DCFH-DA Staining to Detect Cellular Reactive Oxygen Species (ROS) Levels. After reaching the incubation time, cells were washed with PBS, fixed with 4% paraformaldehyde for 15 min, washed with PBS, and sealed with sheep serum for 20 min at room temperature, followed by the addition of primary antibody (1:100) for OPN and incubation overnight at 4 °C. The cells were washed three times with PBS, incubated for 30 min at 37 °C with iFluor™ 488 secondary antibody (1:800), washed three times with PBS, and incubated for 5 min at room temperature with DAPI staining solution. The expression of OPN was then observed by laser confocal fluorescence microscopy.

##### Qualitative and Quantitative Observation of Cell Adhesion

**Cell preparation:** The seed plates and experimental groups are the same as in Section Cell Protection by Polysaccharides. After reaching the incubation time, the cells were washed twice with cold PBS and incubated at 4 °C for 0.5 h to inhibit crystal endocytosis, followed by the addition of 200 μg/mL FITC-COM at 4 °C for 1 h. After reaching the incubation time, the cells were washed twice with cold PBS.

**Confocal microscopy for qualitative observation of cell adhesion:** Prepared cells were stained with DiI for 10 min, fixed with 4% paraformaldehyde for 15 min, and stained with DAPI for 5 min. The crystal adhesion was observed by laser confocal microscopy.

**Quantitative detection of the proportion of cells adhering to the crystals:** After the cells were digested with trypsin and resuspended with PBS, the average fluorescence intensity and the proportion of cells adhering to the crystals were detected by flow cytometry. The cells with FITC signals could be regarded as cells with crystal adhesion.

#### 4.2.7. Detection of Cell Death and Apoptosis

##### Qualitative Detection of Cell Death by AO/EB Double Staining

The seed plates and experimental groups are the same as in Section DCFH-DA Staining to Detect Cellular Reactive Oxygen Species (ROS) Levels. After the incubation time was reached, the medium was aspirated, and the cells were washed twice with PBS. Each well had 1 mL of PBS and 40 μL of the AO/EB working solution added, and they were placed at room temperature for 5 min and then observed under a fluorescence microscope. The maximum excitation wavelength and emission wavelength of the AO-DNA complex were 488 nm and 515 nm, respectively. The maximum excitation and emission wavelengths of EB-DNA complexes are 518 nm and 605 nm, respectively.

##### Annexin V/PI Double Staining for Quantitative Detection of Apoptosis

Cell seeding plates and experimental groupings were the same as in Section DCFH-DA Staining to Detect Cellular Reactive Oxygen Species (ROS) Levels. After the incubation time was reached, the cell culture medium was collected and set aside, the cells were washed with PBS, cells were digested by adding EDTA-free trypsin and incubated at room temperature until the cells could be blown off by gentle blowing, the trypsin was aspirated, the collected cell culture medium was added, the cells were blown and centrifuged, the supernatant was discarded, and the cells were collected. Furthermore, 500 μL of Binding Buffer was added to suspend the cells, 5 μL of Annexin V-FITC was added and mixed well, and 5 μL of PI staining solution was added and mixed well; this was incubated for 10 min at room temperature in the absence of any light, centrifuged, resuspended in PBS, and immediately assayed by flow cytometry.

#### 4.2.8. Statistical Analysis

All experimental data are shown as the mean ± standard deviation ($\bar{x}$ ± SD) of three parallel groups. A one-way analysis of variance (One-way ANOVA) test was performed using IBM SPSS Statistics 26 statistical analysis software (IBM Corp. Released 2019. IBM SPSS Statistics for Windows, Version 26.0. IBM Corp, Armonk, NY, USA) to analyze the differences in mean value between the experimental group and the control group. *p* > 0.05 indicates no significant difference. * *p* < 0.05 was considered significant and ** *p* < 0.01 indicated a highly significant difference.

## 5. Conclusions

DLP and SDLP exhibit a potent capacity to counteract nano-COM-induced oxidative stress and ER stress within renal tubular epithelial cells. In comparison to the injury group, the DLP and SDLP protection groups demonstrated marked enhancements in cell viability, a reduction in cellular ROS levels and mPTP opening, and a decline in the expression of ER Ca^2+^ ions, ER stress signature proteins, CHOP, and Caspase 12. Moreover, the levels of the p38 MAPK protein, apoptosis rate, cell surface adhesion molecule OPN, and crystal adhesion to the cell surface were all mitigated. Notably, the effect of SDLP outshone that of DLP. Collectively, these findings underscore that SDLP holds the potential to shield HK-2 cells from nano-COM crystal-induced apoptosis and to curtail crystal–cell adhesion dynamics by effectively suppressing oxidative stress and ER stress. Consequently, SDLP emerges as a promising contender for mitigating cell damage caused by crystal interactions and for thwarting the recurrence of stone formation.

## Data Availability

Data are contained within the article.
